# Nursing in Rural Districts

**Published:** 1898-03-19

**Authors:** 


### NURSING IN RURAL DISTRICTS.

How best to organise a system of nursing for a country district is a matter of such great importance that we need not be surprised that many schemes should be put forward. Nor need we be surprised to find that, from want of definite knowledge as to the legal responsibilities of the various public bodies which at present have to do with the sick poor, mistakes should now and then be made. It is evident, however, that there is a real desire in some places to set up a complete system of nursing which shall cover the whole field, and be available both for those who can pay and those who cannot. We recently received a letter from Dr. A. H. Frere, of Stradbroke, Suffolk, on this subject. He wrote as follows:—

"There is a probability of a certain workhouse being opened, and I am anxious to start a nursing scheme for this, a wide agricultural district. I want to know if the workhouse can be made the basis of our operations. If so should the nursing scheme pay the Guardians so much a year to be allowed to take cases (not infectious) there, or should it be worked in some other way? We are a very long way from any hospital, and a cottage or workhouse hospital is much needed. There are many small farmers and club patients who could often be better treated there."

In a further communication he says:—

"I saw it suggested in the *British Medical Journal* that the base of a nursing scheme might be either a cottage hospital or a workhouse hospital. As you say, the former is simple enough. I want to know how to organise a nursing scheme in connection with the sick wards of a workhouse. And here my chief difficulty is the financial one. Could the District Council throw open these wards to the district, the patient paying so much a week? Or could the nursing scheme pay the Guardians so much a year to be allowed to send cases into these sick wards. I am not sure what the Local Government Board would sanction in this matter. I should aim at having a trained nurse at the workhouse, a district nurse under her, and should then train some person in each place to act as village nurse under the district nurse. I want to know if all this should be worked out by the District Council, or should others organise a nursing scheme and then get the Guardians to help us by allowing us to make the workhouse the base of operations."

We are afraid, however, that neither our correspondent nor the *British Medical Journal* fully realise the distinction between the duties and legal powers of the Poor Law Guardians and those of the local sanitary authority; or, if they realise it, do not consider it of great importance. The Local Government Board, however regard the distinction as one of vital importance, and act accordingly. The Guardians are appointed to administer the Poor Law and to look after the destitute poor, whether indoor or outdoor, and to relieve destitution either in a workhouse or by way of outdoor relief. They cannot legally admit to their workhouse any but paupers, and they cannot spend their funds except on behalf of the destitute. If a nursing institute were privately organised and privately supported and administered in their neighbourhood the Guardians would have full power to subscribe or contribute to it, under section 10 of the Poor Law Act, 1879, if by so doing they could avail themselves of the services of the institute in nursing their sick poor; but they could not allow their workhouse or infirmary (if in use and required by themselves) to be used also for the nursing of non-pauper cases. Moreover, if there were no legal objections, it is not likely that non-paupers would ordinarily be found willing to risk the stigma of pauperism by being removed to the workhouse or the Poor Law infirmary. In the present case there is, however, an empty workhouse in Dr. Frere's neighbourhood, to which we will refer presently.

Then, as regards the District Council or local sanitary authority, who are responsible for the public health of their district as a whole, and who with that object have to administer and enforce the Public Health Acts. They have full power under section 131 of the Public Health Act, 1875, to "provide for the use of the inhabitants of their district hospitals or temporary places for the reception of the sick," &c., and to provide all necessary nursing, medical attendance, medicine, food, &c,, for the treatment of patients in such hospitals. But they are not directly empowered to provide nurses for the treatment of persons in their own homes; indeed, it is very doubtful if even by "juggling" they can legally do so. If a district council does do so they have to risk the possibility of the district auditor disallowing or surcharging the payments and cost in the accounts. As a rule district councils restrict their functions to dealing with infectious diseases, and providing hospital accommodation for such diseases only. But if they are so minded (and this mental attitude is quite the exception), it is held to be open to them, under the Public Health Act, to provide hospital accommodation for accident cases and general "sick." As an illustration, it may be mentioned that Mr. Passmore Edwards recently offered a sum of money to provide an accident hospital at Tilbury Docks, if the local authority (the Grays Thurrock Urban District Council) would undertake to maintain it. The Local Government Board advised the District Council that it was competent to them to do so if they chose, and they have acted accordngly. The Widnes Town Council have also provided an accident hospital at the cost of the rates, and so have one or two other authorities.

But surely the most satisfactory method of effecting what Dr. Frere wishes is to form a nursing association or local committee, supported mainly by voluntary subscriptions and the payments (according to means) of hose who will employ the nursing resources of the association or committee, and to secure at the same time the financial support of the Poor Law Guardians, and also, where practicable, of the District Council or sanitary authority. Such an association should, of course, if possible, have a cottage hospital to work from and in connection with; and Dr. Frere does not say that this is not practicable in his district. As a matter of fact, there is at Hoxne an empty workhouse (possibly the workhouse to which Dr. Frere incidentally refers in his letter), as the Guardians of the Hoxne Union board out their indoor paupers in the workhouse of the adjoining Hartismere Union: and last year a movement was started for converting that building, or part of it, into an infirmary to commemorate the Queen's Diamond Jubilee. But the Jubilee fever seems to have cooled down without anything definite having been done. The Local Government Board were appealed to, and promised that they would be prepared to consider any application which might be made to them for their assent to the letting of the disused building in question, or part of it, for use by a nurse appointed by the contemplated private nursing association. The Board, however, pointed out that the furnishing of the rooms should be undertaken by the association appointing the nurse, and not by the Guardians; but that the Guardians might, with the Board's consent, subscribe to the funds of the association. Is there not here, therefore, ready to hand, the germ of a useful arrangement or association? Could not funds be got together to purchase this empty building, and fit it up, or part of it, as a cottage hospital or infirmary, which could be supported partly by subscriptions from the Guardians, partly by aid from the District Council, and partly by patients' payments and voluntary subscriptions? Or, if at first the money to purchase the building outright cannot be procured, might it not usefully be hired at a nominal rent from the Guardians (who appear to be willing to let and to assist), and be partially fitted up as an infirmary? But if success is to be attained by any such scheme it must be kept clear of all suspicion of pauperism of Poor-Law taint, and must not be managed or controlled by the Guardians as such.

